# Activity of *Cinnamomum* osmophloeum leaf essential oil against *Anopheles gambiae* s.s

**DOI:** 10.1186/1756-3305-7-209

**Published:** 2014-05-02

**Authors:** France P Mdoe, Sen-Sung Cheng, Shandala Msangi, Gamba Nkwengulila, Shang-Tzen Chang, Eliningaya J Kweka

**Affiliations:** 1 Department of Zoology and Wildlife Conservation, College of Natural and Applied Sciences, University of Dar-es-salaam, PO Box 35165 Dar-es-salaam, Tanzania; 2 Experimental Forest, National Taiwan University, Nan-Tou 557, Taiwan; 3 Division of Livestock and Human Diseases Vector Control, Tropical Pesticides Research Institute, Ngaramtoni, Off Nairobi Road, PO Box 3024 Arusha, Tanzania; 4 School of Forestry and Resource Conservation, National Taiwan University, No. 1, Sec.4, Roosevelt Road, Taipei 106, Taiwan; 5 Department of Medical Parasitology and Entomology, Catholic University of Health and Allied Sciences, PO Box 1464, Mwanza, Tanzania

## Abstract

**Background:**

The increasing status of insecticide resistant mosquitoes in sub-Saharan Africa is a threatening alert to the existing control efforts. All sibling species of *An. gambiae* complex have evolved insecticide resistance in wild populations for different approved classes of the insecticides currently in use in the field. An alternative compound for vector control is absolutely urgently needed. In this study, the larvicidal activity and chemical composition of the *Cinnamomum osmophloeum* leaf essential oils were investigated.

**Methods:**

*C. osmophloeum* leaf essential oils were extracted by hydrodistillation in a Clevenger-type apparatus for 6 hours, and their chemical compositions identified using GC-MS. These oils were evaluated against *An. gambiae* s.s. in both laboratory and semi-field situations. The WHO test procedures for monitoring larvicidal efficacy in malaria vectors were used.

**Results:**

The composition of *C. osmophloeum* leaf essential oil has been found to have 11 active compounds. The most abundant compound was *trans*-cinnamaldehyde (70.20%) and the least abundant was caryophyllene oxide (0.08%). The larvicidal activity was found to be dosage and time dependant both in laboratory and semi-field environments with mortality ranging from 0% to 100%. The LC_50_ value was found to vary from 22.18 to 58.15 μg/ml in the laboratory while in semi-field environments it was 11.91 to 63.63 μg/ml. The LC_90_ value was found to range between 57.71 to 91.54 μg/ml in the laboratory while in semi-field environments was 52.07 to 173.77 μg/ml. Mortality ranged from 13% to 100% in the laboratory while in semi-field environments it ranged between 43% to 100% within mortality recording time intervals of 12, 24, 48, and 72 hours.

**Conclusions:**

The larvicidal activity shown by *C. osmophloeum* leaf essential oil is a promising alternative to existing larvicides or to be incorporated in integrated larval source management compounds for *An. gambiae* s.s control. The efficacy observed in this study is attributed to both major and minor compounds of the essential oils.

## Background

Malaria vector species insecticide resistance status has been a drawback from the existing control tools. Insecticide resistance has been reported for the main malaria vectors, *An. gambiae* s.s, *An. arabiensis* and *An. funestus*[1-5]. Mosquitoes play a major role in the transmission of public health diseases such as malaria, dengue fever and arboviruses diseases which causes high mortality and morbidity for human populations globally [6,7]. The classes of insecticides which are used for indoor residual spray (i.e., organophosphates, carbamate, organochlorides, and pyrethroids) for net treatment and for larvicides (carbamates) have been reported to have reduced mortality [1-3,5,8]. Currently there is a need for the new class of insecticide molecules which will be incorporated in integrated vector management to reduce the insecticide resistant problem. In recent years, there is renewed interest on the plant kingdom to produce bio-pesticides. In malaria endemic areas several plant species have been used to extract essential oils for different insecticidal activities be it for crop pests control [8-10] or animal and human medicine and biting pests control [11].

Arthropod control plant based insecticides have been used since ancient times [12,13]. Malaria vector control has received serious attention in control using plant material. Plant materials have been used as repellents by burning or hanging whole plants indoors [11,14-16] by having selected repellent plants around houses [16]. In advancement of technology plant essential oil extraction has been through distillation and used as plant repellents against mosquito and other biting midges [17,18]. In invention of GC-MS it has been possible to separate individual components of plant essential oils and test their repellent, larvicidal, ovicidal or adulticidal efficacy for each single compound [19].

Family Lauraceae consists of trees of economic importance. The genus *Cinnamomum* comprises of 250 species distributed in Asia and Australlia [20]. *C. osmophloeum* Kaneh has been of great interest in research due to its chemical constituents being similar to those of *C. cassia* bark oil [21]. Cinnamon is an endangered tree that grows in Taiwan at an elevation of 400 to 1500 m in natural hardwood forest. In previous research findings, essential oils extracted from leaves of *C. osmophloeum* have antiviral, antifungal, and antibacterial activities [22,23]. Also the essential oil from the leaves of *C. osmophloeum* has been found to have the strongest mosquito larvicidal activities against different mosquito species [24,25]. It is of interest to understand if the essential oil from leaves of *C. osmophloeum* possesses action against Afrotropical malaria vectors *An. gambiae* s.s. Therefore, it was the aim of this study to assess the chemical composition of essential oils from leaves of *C. osmophloeum* and its larvicidal activity against *An. gambiae* s.s mosquito larvae.

## Methods

### Plant material and essential oil extraction

Mature leaves of *C. osmophloeum* were collected in July, 2008 from the Da-Pin-Ting of the Taiwan Sugar Farm, the location is situated in Nantou County of central Taiwan. The species were identified by Mr. Yen-Ray Hsu of the Taiwan Forestry Research Institute and the voucher specimen (COL027) was deposited at the laboratory of wood chemistry, School of Forestry and Resource Conservation, National Taiwan University. The sample (200 g each), in triplicate, was subjected to hydrodistillation in a Clevenger-type apparatus for 6 hours [26], followed by determination of oil contents. The leaf essential oil obtained was separated from the aqueous phase and stored in airtight containers at 4°C prior to further analysis.

### GC-MS analysis

Analyses of volatile constituents were determined using a Polaris Q Ion Trap GC/FID/MS^n^ system (Thermo, USA), equipped with a fused silica column (30 mm × 0.25 mm i.d.) and coated with 5% phenyl-methylpolysiloxane using a DB-5 ms (df = 0.25 μm) (Agilent J & W Scientific). The temperature program used for the analysis was as follows: initial temperature at 80°C, held for 1 min, ramped at 4°C/min to 200°C and held for 5 min. Helium was the carrier gas at 1.0 ml/min; the sample (1.0 μl, 1/100, v/v, in ethyl acetate) was injected in the split mode (1:10). The mass spectrometer was equipped with a Polaris Q mass selective detector in electron impact (EI) ionization mode (70 eV). The ion source temperature and the injector temperature were set at 230°C and 270°C, respectively. The sector mass analyzer was set to scan from 50 to 450 amu every 0.5 s. The Kovats retention indices were calculated for all volatile constituents using a homologous series of *n*-alkanes C_9_-C_17_ on DB-5 ms column. Quantification was performed using percentage peak area calculations and the identification of individual components was done using the Wiley/NBS Registry of Mass Spectral Database and NIST MS Search 2.0, the literature [27], and several authentic reference compounds. The relative concentration of each compound in essential oil was quantified according to the peak area integrated by the analysis program.

### Larval rearing for experiments

Eggs laid were incubated for 48 hours in an insectary incubator at a temperature of 27 ± 2°C and a relative humidity of 78 ± 2%. Immediately after 48 hours of incubation filter papers were washed on a rearing pan. The first instar larvae were provided with yeast. Second instars onwards were fed with tetramin fish food 0.003 gm per larvae. Third instar larvae were used for experiments in evaluating the larvicidal action of the leaf essential oil of *C. osmophloeum.*

### Larval bioassays in the laboratory

A number of cups (diameter 10 cm, depth 10 cm with capacity of 100 mls) were prepared in six replicates for each treatment dosage with a control, and each replicate received 20 third instar larvae, as well as control. One control contained normal laboratory larval rearing water while the other was an aqueous solution of 0.50% of dimethyl sulfoxide (DMSO) that was used as dispersing medium for the essential oil and exposure to larvae for treatment. The number of serial dilutions made for dosages effective enough to cause mortality were as described in WHO larval bioassay protocol [28]. The test was performed by serial dilutions, 200, 100, 50, 25, and 12.5 μg/ml, using normal laboratory larval rearing water. Larvae were not provided with food and mortality recordings were taken at 12, 24, 48, and 72 hours for both treatments and control. The dead and moribund larvae were all considered as dead.

### Larval bioassay in the semi-field

Semi-field environment structures used in this study were similar to those described in previous studies [29]. The semi-field evaluation protocol was adopted from the WHO protocol [28]. The same dosages used in the laboratory were evaluated in the semi-field. A series of cups (with diameter of 10 cm and depth10 cm, with capacity of 100 mls) were prepared in six replicates for each control and treatment, and each replicate received 20 third instar larvae. Controls, one had normal laboratory larval rearing water and the other had an aqueous solution of 0.50% of DMSO that was used as dispersing medium for the essential oil and exposure of larvae to treatment. Other procedures were performed similar to the larval bioassays in the laboratory.

### Statistical analysis

The Scheffe multiple comparison procedure of SAS statistical program was used to analyse mosquito larvicidal activity of *C. osmophloeum* leaf essential oil (*P* < 0.05). All results were obtained from four independent experiments and expressed as mean ± SD.

Mortality was recorded after 12, 24, 48, and 72 h of exposure, during which no food was given to the larvae. Percent mortality was corrected for control mortality using Abbott’s formula [30], and the results were plotted on log/probability paper using the method developed by Finney [31]. Toxicity and activity were reported as LC_50_ and LC_90_, representing the concentration in μg/ml that caused 50 and 90% larval mortality, respectively, in 12, 24, 48, and 72 hours.

## Results

### Chemical composition

In GC-MS chemical analysis of the *C. osmophloeum* leaf essential oils have found 11 compounds with *trans*-cinnamaldehyde as the highest abundant compound with 70.20% and caryophyllene oxide found to have the least amount of 0.8%. The other 9 compounds occurred in different composition percentages (Table 1). The yield of essential oils from *C. osmophloeum* leaves was 44.47 ± 0.68 mls/Kg and composition of 2.82 ± 0.09% (w/w).

**Table 1:** **Constituents of leaf essential oil from *Cinnamomum osmophloeum***

**NO**	**RT**^**a**^	**Compounds**	**Area, %**	**KI**^**b**^
1	6.45	Benzaldehyde	0.45	965
2	12.61	Benzenepropanal	0.82	1164
3	13.05	2-Methyl benzofuran	0.14	1176
4	13.83	4-Allylanisole	0.17	1197
5	14.51	*cis*-Cinnamaldehyde	0.33	1219
6	16.31	*trans*-Cinnamaldehyde	70.20	1273
7	16.74	L-Bornyl acetate	0.48	1285
8	18.90	Eugenol	0.10	1351
9	21.15	*trans*-β-Caryophyllene	0.18	1419
10	21.92	*trans*-Cinnamyl acetate	27.05	1444
11	26.09	Caryophyllene oxide	0.08	1580
**Total identified**			100.00	

^a^Retention time (min).

^b^Kovats index relative to *n*-alkanes (C_9_-C_17_) on a DB-5 ms column.

### Larval bioassays in the laboratory

From the range of doses used, the mortality effect from 12 to 72 hours of observation was found to be dosage dependant. In each mortality observation time, the lowest dosage had lowest mortality while the highest dosage had the highest mortality (Table 2). After 72 hours the mean percentage mortality ranged from 13 to 100% for *An. gambiae* s.s. larvae (Table 2). The interval observations (12, 24, 48, and 72 hours) in each concentration among laboratory experiments had mortality responses that varied with time (Table 2). The dosage of essential oil required to kill 50% (LC_50_) and 90% (LC_90_) of the larvae varied with time of exposure (Table 3).

**Table 2:** **Larvicidal effect of *Cinnamomum osmophloeum *** **leaf essential oil against *Anopheles gambiae *** **s.s in different time intervals and concentrations in the laboratory**

**Time**	**200**	**100**	**50**	**25**	**12.5**	**Equation**	**R**^**2**^	**LC**_**50**_	**LC**_**90**_
0 h	0	0	0	0	0				
12 h	100	100	43	3	0	y = 1.1979x - 19.652	0.9873	58.15	91.54
24 h	100	100	80	19	1	y = 51.648Ln(x) - 134.15	0.9477	35.36	76.70
48 h	100	100	95	44	9	y = 46.743Ln(x) - 104.66	0.9264	27.35	64.36
72 h	100	100	97	68	13	y = 41.838Ln(x) - 79.672	0.8615	22.18	57.71

**Table 3:** **LC**_**50 **_**and LC**_**90 **_**values of leaf essential oil from *Cinnamomum osmophloeum *** **in laboratory and semi-field environments against *Anopheles gambiae *** **s.s**

**Time**	**LC**_**50 **_**(μg/ml)**		**LC**_**90 **_**(μg/ml)**	
	**Laboratory**	**Semi-field**	**Laboratory**	**Semi-field**
12 h	58.15	63.63	91.54	173.77
24 h	35.36	42.46	76.70	131.07
48 h	27.35	22.37	64.36	94.08
72 h	22.18	11.91	57.71	52.07

### Larval bioassay in the semi-field

The mortality trends observed in the semi field experiments were similar to that in laboratory assays for mortality been dosage dependance in every observation time. Mortality rate was higher in semi-field environments compared to those observed in the laboratory, it ranged between 43% in 12.5 μg/ml to 100% in 200 μg/ml (Table 4). The dosage of essential oil required to kill 50% (LC_50_) and 90% (LC_90_) of the larvae varied with time of exposure, it was observed to be higher than in the laboratory (Table 3) (12, 24, 48, and 72 hours) each concentration among semi-field experiments had mortality responses that varied with time (Table 3).

**Table 4:** **Larvicidal effect of *Cinnamomum osmophloeum *** **leaf essential oil against *Anopheles gambiae *** **s.s in different time intervals and concentrations in semi-field environments**

**Time**	**200**	**100**	**50**	**25**	**12.5**	**Equation**	**R**^**2**^	**LC**_**50**_	**LC**_**90**_
0 h	0	0	0	0	0				
12 h	100	80	18	4	0	y = 39.818Ln(x) - 115.37	0.89	63.63	173.77
24 h	100	92	48	32	7	y = 35.49Ln(x) - 83.039	0.96	42.46	131.07
48 h	100	99	81	58	24	y = 27.844Ln(x) - 36.526	0.91	22.37	94.08
72 h	100	100	96	79	43	y = 27.123Ln(x) - 17.204	0.87	11.91	52.07

## Discussion

The findings of this study have shown that the essential oils of *C. osmophloeum* leaves have potential in inducing larval mortality both in semi-field and in laboratory environments. The mortality in the laboratory ranged from 13 to 100% while for semi-field ranged from 43 to 100% for dosages ranging from 12.5 μg/ml to 200 μg/ml respectively. The mortality increase was observed in semi-field trials, this might be attributed with the breakdown of compounds into secondary metabolites, which are more efficient when exposed to sunlight. The same trend was revealed when the plant essential oils were evaluated in a semi-field environment where experiments are exposed to sunlight and more mortality was observed than in the laboratory [29]. In other experiments mortality was observed to decrease when experimental material was exposed to sunlight in semi-field environments [29]. This shows that, the plant phyto-compounds should be assessed enough before exposed to sunlight for whether its secondary metabolites increase or decrease larval mortality. The larvicidal efficacy of the essential oils in both laboratory and semi-field experiments were shown to decrease with time as shown by LC_50_ and LC_90_ values in Table 3.

The mortality response caused by *C. osmophloeum* leaf essential oil to *An. gambiae* s.s larvae might have been influenced highly by *trans*-cinnamaldehyde and which occurred in higher proportion that others, but also, *cis*-cinnamaldehyde might have a role in inducing mortality as well. In other experiments where the active compounds of *C. osmophloeum* leaf essential oil were evaluated singly, *trans*-cinnamaldehyde and *cis*-cinnamaldehyde induced the highest mortality against *C. quinquefasciatus* and *A. subalbtus* larvae at a minimal value of LC_50_ and LC_90_[24]. LC_50_ and LC_90_ values observed in the current study for mortality of *An. gambiae* s.s larvae have been shown to be higher than in previous studies reported by Cheng and others [24], this might be attributed to hindrance factors from other active compounds in the essential oils evaluated. Evaluation of the active ingredient of the essential oils should be done singly to avoid miss matching of results with previous studies.

The screening of products from these leaf essential oils for larvicidal activity on *An. gambiae* s.s larval mosquitoes might lead to the most efficient compounds for the effective use in the larval sources treatment with residual effect against *An.gambiae* s.s larvae. The uses of synthetic insecticides for routine vector control have resulted in emergence of increase of resistance selection pressure among disease vectors [1-5]. This has increased the interest on searching for environmentally friendly, biodegradable, low-cost, and indigenous methods for vector control, which can be used without risk to none targeted organisms in communities [17,32]. The control of mosquito borne diseases can effectively achieve abatement which results in reduction of mosquitoes- human contact rates.

In this study, the laboratory and semi-field findings showed highest efficacy in mortality at lower dosages for the *An. gambiae* s.s. larvae (Tables 2 and 3). In the laboratory, mortality was dosage dependent, while in the semi-field, where other variables such as wind and sunlight were present, there was increased toxicity in lower dosages which increased mortality impact on larvae which was greater than the effect observed within laboratory experiments (Tables 2 and 3). The higher mortality in the lower dosages in the semi-field might be attributed to the degradation of compounds due to exposure to sunlight hence inducing more toxicity. The exposure of plant extracts to sunlight causes molecules to degrade to secondary metabolites which are thought to be the cause of the higher mortalities in semi-field experiments [29].

This essential oil may be of great value in complementing other compounds which are losing their efficacy against vectors, such as pyrethroids and others which are considered none-biodegradable and environmental pollutants such as 1,1,1-trichloro-2,2-bis(*p*-chlorophenyl) ethane (DDT) [33]. From other studies, some phytochemicals have acted as general toxicants against adult as well as larval stages of mosquitoes, while others interfere with growth and development (growth inhibitors) or with reproduction (chemosterilent) or produce olfactory stimuli acting as repellent or attractant [34]. There is renewed interest in the plant based resources for disease vector control bio-pesticides due to increased resistance for the existing selected insecticides in different vectors [1-5,7]. To date larval source management or control have shown a great impact when done alone or integrated with others tools such as indoor residual spray or insecticide treated nets, control impact has been observed in other areas [35]. This is because larval habitat treatment is more localized in time and space resulting in effective control as larvae cannot move from one habitat to another. In tropical countries, plants are known to possess larvicidal, ovicidal, and adulticidal activities [11,29].

The results of our current study in larvicidal properties of the essential oil of leaves of *C. osmophloeum* have created the necessity of investigating particular larvicidal activities of each chemical compound in this essential oil and drive their larvicidal activity potential. Further detailed study on the isolated active ingredients responsible for the larvicidal activity from these plant leaf essential oils may create opportunities for the development of an environmentally safe botanical insecticide for the control of mosquitoes at different stages of their life cycle.

Malarial transmission is much more difficult to control in Africa as compared to most other places because of a complex ecological system, it can be effectively achieved for targeting larval habitats. Larval source management strategies have proved to be infective in contributing to malaria eradication in other countries such as Brazil [36]. In that way, the leaf essential oil of *C. osmophloeum* a renowned natural source of larvicides could be used for the control of the African malaria vector mosquito, *An. gambiae* s.s*.*

## Conclusion

The findings of this study have shown high mortality of *An. gambiae* s.s. third instar larvae caused by *C. osmophloeum* leaf essential oils was dosage and time dependant in both laboratory and semi-field trials. Further evaluations are ongoing for the efficacy of *C. osmophloeum* leaf essential oil adulticidal effects on wild populations of *An. gambiae s.s.*

## Competing interests

Authors declare to have no competing interest in this work.

## Authors’ contributions

EJK, FPM, and STC conceived and designed the study. STC, SSC, and EJK carried out the data analysis and interpretation. FPM and SM did data collection and entry. FPM, EJK, and STC wrote the manuscript. EJK, GN, SSC, and STC revised the manuscript critically. All authors approved the final version for submission.
